# Weather and Health Symptoms

**DOI:** 10.3390/ijerph15081670

**Published:** 2018-08-06

**Authors:** Mihye Lee, Sachiko Ohde, Kevin Y. Urayama, Osamu Takahashi, Tsuguya Fukui

**Affiliations:** 1 Graduate School of Public Health, St. Luke’s International University, Tokyo 104−0045, Japan; saohde@luke.ac.jp (S.O.); kevura@luke.ac.jp (K.Y.U.); otakahas@luke.ac.jp (O.T.); fkts@luke.ac.jp (T.F.); 2 Department of Social Medicine, National Center for Child Health and Development, Tokyo 157−8535, Japan; 3 St. Luke’s International Hospital, Tokyo 104−8560, Japan

**Keywords:** weather and physical symptoms, pain, joint pain, headache, cough, temperature, humidity, depressed mood

## Abstract

Weather affects the daily lives of individuals. However, its health effects have not been fully elucidated. It may lead to physical symptoms and/or influence mental health. Thus, we evaluated the association between weather parameters and various ailments. We used daily reports on health symptoms from 4548 individuals followed for one month in October of 2013, randomly sampled from the entirety of Japan. Weather variables from the monitoring station located closest to the participants were used as weather exposure. Logistic mixed effects model with a random intercept for each individual was applied to evaluate the effect of temperature and humidity on physical symptoms. Stratified analyses were conducted to compare weather effects by sex and age group. The lag day effects were also assessed. Joint pain was associated with higher temperature (1.87%, 95% CI = 1.15 to 2.59) and humidity (1.38%, 95% CI = 0.78 to 2.00). Headaches was increased by 0.56% (95% CI = −0.55 to 1.77) per 1 °C increase in the maximum temperature and by 1.35% per 1 °C increase in dew point. Weather was associated with various physical symptoms. Women seem to be more sensitive to weather conditions in association with physical symptoms, especially higher humidity and lower temperature.

## 1. Introduction

Weather affects the daily lives of humans. Furthermore, many individuals claim that certain weather conditions aggravate or alleviate their physical symptoms or mental health [1,2]. However, these arguments need to be given full systematic consideration in a comprehensive manner.

There have been several studies that examined an association between weather variables and physical ailments. For instance, Ferreira et al. [3] evaluated if exacerbation of knee pain is associated with weather in 345 patients with knee osteoarthritis, and found no associations. Dorleijn et al. [4] observed an association between barometric pressure/relative humidity and perceived osteoarthritis symptoms in 222 patients with hip osteoarthritis; however, they concluded that the effect size is ignorable in a clinical sense. Yang et al. [5] examined the relationship between headache and temperature in 66 migraine patients in Taipei, Taiwan and have shown that perceived sensitivity to temperature is associated with higher headache incidences. A systematic review [6] assessed nine studies on weather and rheumatoid arthritis and reported inconsistent results across the studies. Another review [7] stated that recently published studies tend to confirm the association between weather and osteoarthritic pan. Across studies, evidence is still inconsistent and thus, inconclusive.

Weather is also believed to affect mood. In their influential study, Schwarz and Clore [8] claimed that weather affects mood, which alter humans’ judgement on their life satisfaction. However, subsequent studies that assessed mood as a main outcome produced diverse results. Klimstra et al. [9] found a minimal or null association between weather and mood. Kööts et al. [10] found a weak association of mood and fatigue with temperature and sunlight. Keller et al. [11] found higher temperature and barometric pressure was related to better mood in the spring. As with the studies on weather and physical ailments, prior studies on mood have yielded mixed results.

McAlindon [12] described the main limitations of previous studies on weather effects on health symptoms: awareness of the study hypothesis by participants, small sample size, and lack of geographical diversity. He noted that the disclosure of study objective to study participants may increase their sensitivity or perception of pain. He also pointed out that previous studies had been based on relatively small sample sizes, which had limited the ability to detect modest effects of weather and failed to consider effect modification by subgroups. Therefore, the opportunity to identify the vulnerable populations with respect to the effect of certain weather conditions on health symptoms has been limited. Lastly, a selection of one local area for investigation can lead to less variation in weather distribution.

Also, the majority of studies aforementioned have focused on patients with specific diseases, which restricts the ability to generalize the result to the general population. Furthermore, since some physical symptoms, such as headache, tend to repeat within the same individual, their frailty should be taken into account as well. A cross-sectional study design cannot fulfill this condition; however, a longitudinal study that repeatedly measures symptoms of individuals for a period of time is capable of adjusting for within person correlation. Therefore, studies that address these previous limitations may have the ability to offer robust evidence to a currently inconsistent literature. 

The Health Diary Study [13] conducted in Japan in October 2013, has the ability to address this research question while overcoming several of the limitations of previous studies. The original purpose of the study was to evaluate the health care seeking behaviors for different types of ailments and physical/mental symptoms. In doing so, over 4500 individuals were randomly recruited from across Japan and were followed for one month during which time they were asked to keep a daily health diary. The data collection effort also enabled a unique opportunity to address the topic related to the influence of weather patterns on health-related symptoms which were unaffected by the types of limitations described above.

In the current study, we evaluated the association between weather parameters and various ailments encompassing physical symptoms and mental health in a population of over 4500 individuals recruited across Japan who were followed daily for one month. Specifically, we hypothesized that physical and psychological symptoms are associated with the variation in weather and there are differences in perceived pain between sex and age groups.

## 2. Materials and Methods 

### 2.1. Data

#### 2.1.1. Japanese Health Diary Study

Fukui et al. [13] reported on the ecology of medical care in Japan and evaluated the relationship between health symptoms and patterns of health care utilization. In their study, they used a survey company that maintained a roster of 79,749 individuals (37,643 men and 42,106 women). Among them, 19,633 agreed to participate in the study. Finally, 5000 individuals were selected according to population-weighted random sampling to ensure that the cohort represents the general population of Japan.

At the beginning of the study, participants were asked to complete and return a baseline questionnaire. Content of the questionnaire included demographics, socio-economic status, and lifestyle factors.

In addition, they were asked to keep a daily record of any physical symptoms they had and measures they took to cope with the corresponding symptom (referred to as a daily health diary). Symptoms were stated in a descriptive format. In cases where the participant was a child aged less than 13 years old, his or her parent answered the baseline questionnaire and completed the health diary. They were followed from 1 October through October 31. A manual was also given to each participant to provide instruction on answering the questionnaire and health diary, and phone calls were made every week to ensure adherence. As a result, 4548 individuals completed the health diary corresponding to a response rate of 91%. After health diaries were collected, narrative symptoms were coded by staff. Researchers and research assistants reviewed and cross-checked those narrative symptoms in compliance with the International Classification of Primary Care Second Edition (ICPC-2) [14].

Written informed consent was obtained and the study was approved by the Research Ethics Committee of the St. Luke’s International Hospital. All procedures were performed in accordance with relevant guidelines and regulations.

#### 2.1.2. Weather Data

We acquired meteorological data for late September and October 2013 from the Japan Meteorological Agency. Weather variables included average, minimum, and maximum temperature in Celsius, relative humidity in percentage, barometric pressure in hectopascal, daylight hours in hour, precipitation in millimeter, and wind speed in meter per second.

We used the maximum temperature as the temperature indicator and the dew point for humidity on the same day of symptom occurrence. The maximum temperature was more relevant to the actual temperature to which individuals were exposed under their diurnal activities and the outdoor environment. With respect to the humidity variable, Wallace and Hobbs [15] described that the relative humidity does not properly represent the absolute amount of water vapor in the atmosphere, nor the human discomfort level. A relative humidity of 70% at cooler temperatures may be considered comfortable for some, but the same humidity level may be intolerable at higher temperatures. Rather, they suggested that the dew point is a more reliable indicator in terms of the moisture content of the air as well as human discomfort. Therefore, we decided to use the dew point as a variable to represent humidity. The dew point is the temperature at which the concentration of water vapor in the air is saturated, and thus forms dew. We calculated the dew point using the following formula [16]

(1) $$\;DPT_{i}={\left(\frac{RH_{i}}{100}\right)}^{\frac{1}{8}}\times \left(112+0.9\times minT_{i}\right)+\frac{minT_{i}}{10}-112\;$$

where *DTP_i_* is the dew point temperature on the *i*th day, *RH_i_* is the relative humidity on the *i*th day, and *minT_i_* is the minimum temperature on the *i*th day.

Weather variables were matched based on the date and the distance. The locations of the weather monitoring stations were provided as latitude and longitude coordinates. We used the zip code number of residence as the study participant’s location. Then, we identified the closest monitoring station to the study participants’ zip code area based on the straight-line distance. We assigned the weather variables of the closest monitoring station on the corresponding date as weather exposure of study participants. ArcGIS 10.5 (Esri, Inc., Redlands, CA, USA) was used to identify the closest weather monitoring station to each zip code of the participant’s address.

### 2.2. Statistical Analyses

Study participants recorded their symptoms on a daily basis, and some of them had recurrent events such as frequent headaches. Hence, the correlation of outcomes within the same individual and status was taken into account. Also, the intrinsic heterogeneity between individuals such as differences in baseline health status needed to be considered. Therefore, we selected the logistic mixed effects model with the random intercept representing each individual.

Linear mixed effects models were devised to model repeated measurements over time accommodating underlying individual differences in their responses [17]. In the model framework, the regression coefficients (the intercept and the slope) are allowed to vary between individuals implying natural heterogeneity in baseline characteristics and trajectories over time. Therefore, the mean response is modeled as a combination of population mean for all individuals (fixed effects) and added individual effects (random effects). The nomenclature (“mixed”) originates from the fact that the model has both terms for fixed effects and those for random effects.

Weather variables display high correlations due to the complex mechanism of the atmospheric process. Therefore, we aimed to select variables most representative of the weather conditions that may affect symptoms while maximizing the model performance. As a result, we ended up with the most relevant weather variables of temperature and humidity. Other weather variables such as barometric pressure or daylight were left out of the model since their effect size were minuscule, did not contribute to the model performance, and displayed high multicollinearity in the presence of the two variables.

In the main model, we regressed the physical symptoms as a function of the maximum temperature and dew point controlling for other covariates. We also included age, sex, BMI, smoking status (current smoker or not), family income (<¥4 million, ≥¥4 million and <¥10 million, and ≥¥10 million), education (≤junior high school, high school or vocational school, ≥university), and alcohol consumption (heavy drinker, if the frequency of drinking > 3~4 days per week or not).

Stratified analyses were conducted to compare weather effects on physical symptoms by sex and age group. Individuals were classified into three age groups: aged 18 years and younger, aged older than 18 and younger than 65, and aged 65 and older. For reliable effect estimation, we excluded symptoms where the number of occurrences was less than or equal to 5.

Previous studies have examined other time windows of exposure up to three days prior to the symptom [12,18]. Therefore, we also examined the lag day effects to assess other time windows that may affect the symptoms more rather than the weather on the same day.

The PROC GLIMMIX procedure was used to fit the model in SAS 9.4 (SAS Institute, Inc., Cary, NC, USA).

## 3. Results

We used data from 4548 participants recorded in October 2013. Table 1 presents the characteristics of the study population. The average age was 44.7 years with a standard deviation of 23.3 years, and 60% of the participants were aged between 18 and 65 years. The number of females was 2365 (52%), and 11% were current smokers. 23.3% were heavy drinkers, and the majority of the participants (65%) had family income between 4 million and 10 million yen. About 50% of the adult population was educated at about high-school or vocational-school level.

The mean distance between the participants’ postal code area and the closest weather monitoring station was 15 km, and the maximum was 67.73 km. The average of daily maximum temperature was 22.8 °C with a standard deviation of 4.6 and the average relative humidity was 72.4% with a standard deviation of 12.7.

Table 2 shows the change in risk of physical symptoms in percentage per temperature change. The risk of joint pain was increased by 1.87% (95% CI = 1.15 to 2.59) per 1 °C increase in the maximum temperature and by 1.38% (95% CI = 0.78 to 2.00) per 1°C increase in dew point (a measure of humidity). The headache risk was increased by 0.56% (95% CI = −0.55 to 1.77) per 1 °C increase in the maximum temperature and by 1.35% (95% CI = 0.40, 2.30) per 1°C increase in dew point. The risk of sneeze was increased when temperature (0.21%, 95% CI = −2.96, 2.62) and dew point (−3.15%, 95% CI = −5.35 to −0.91) both dropped. Itchiness was associated with decrease in humidity (−0.66%, 95% CI = −2.79 to 1.51) whereas eczema was increased when humidity increased (1.33%, 95% CI = −2.65, 5.47), but neither were statistically significant. Eczema was significantly associated with higher temperature (8.54%, 95% CI = 3.47, 13.87). Menstrual cramp was significantly associated with both lower temperature (−6.00%, 95% CI = −8.88 to −3.04) and higher humidity (3.70%, 95% CI = 1.03 to 6.54). Fatigue was associated with higher temperature (1.55%, 95% CI = 0.09 to 3.03) and increase in humidity (1.52%, 95% CI = 0.28 to 2.77). All of the results were statistically significant at the α = 0.05 level.

The association between agitation/anxiety and higher temperature was statistically significant (5.57%, 95% CI = 1.05 to 10.30), whereas its association with higher humidity was not (2.50%, 95% CI = −1.23 to 6.38). Depressed mood showed tendency for an association with lower temperature (−2.71%, 95% CI = −10.98, 6.33) and higher humidity (3.12%, 95% CI = −3.73, 10.45).

Table 3 shows the results from the stratified analyses by sex. There appears to be a tendency for the effect of weather on symptoms in women to be larger than in men. Also, symptoms in women tended to be associated with higher humidity, whereas in men, associations tended to be with lower humidity. With respect to a temperature effect on symptoms, men appeared to be more sensitive to an increase in temperature than women. Depressed mood was associated with higher temperature in men, but associated with lower temperature in women. An increase in humidity was associated with depressed mood in both men and women.

In the examination by age group (Table 4), the aged 65 or older group showed more associations with lower temperature and higher humidity compared to other age groups. The adult age groups showed a stronger association with weather for cramps compared to the group aged 18 or less.

**Figure 1:**
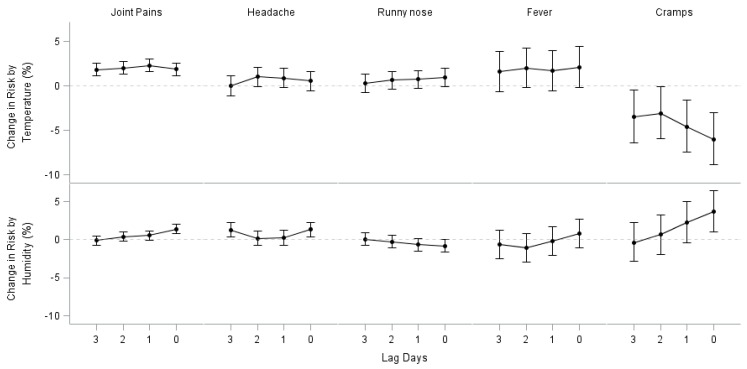
Lag day effects of maximum temperature and humidity.

Analysis by lag time indicated that the time window of exposure for our study is appropriate (Figure 1 and Appendix A). Most of the effect estimates showed the largest effect size on the same day of symptom manifestation.

## 4. Discussion

In this study, we evaluated the association between weather variables and health symptoms in October 2013 among a large population recruited across Japan. We found that substantive numbers of symptoms were affected by weather conditions. Temperature in the form of maximum temperature was the most potent factor, and humidity measured as dew point temperature also appeared to play a significant role. The addition or the replacement of it with pressure did not improve the model outcomes. Likewise, the use of dew point temperature as a humidity indicator outperformed the measure of relative humidity.

In general, temperature showed stronger effects on symptoms than humidity with a few exceptions. It might be explained by homeostasis. The maintenance of a constant body temperature is vital in human survival [19]. Changes in ambient temperature induce immediate reactions in human’s thermal regulation such as cutaneous vasoconstriction for heat loss. Temperature acts as an instant stressor [20]. In comparison, humidity affects perceived temperature rather than temperature itself and thus is more related with a person’s comfort [21]. Headache seemed to be affected more by higher humidity than temperature. The effect of barometric pressure might have been mediated through the humidity variable. Sneezing was affected more by lower humidity than lower temperature. Chill (a sensation of coldness) was the symptom most affected by weather parameters. Its effect size was largest compared with other symptoms, and both coefficients for temperature and humidity were statistically significant. Interestingly, menstrual cramp was one of the main symptoms moderately affected by both lower temperature and humidity. Cold and humid days appeared to affect menstrual cramp. This result is consistent with reports from previous studies where heat therapy appeared to have analgesic effect on menstrual cramp [22,23,24]. Higher temperature was associated with agitation/anxiety while weather did not appear to affect depressed mood. In large, weather effects on human’s mood appear insignificant, which is in concord with the results from previous studies [9,25].

Again, conclusions of previous studies have been inconsistent. Mukamal et al. [26] reported that higher mean ambient temperature increased the risk of headache, and lower barometric pressure increased the risk of non-migraine headaches. Timmermans et al. [18] demonstrated associations between joint pain and higher humidity in older people with osteoarthritis. Strusberg et al. [27] reported low temperature, high atmospheric pressure, and high humidity were significantly correlated with pain in rheumatoid arthritis. Ozeki et al. [28] found that sales of headache medicine increased when average barometric pressure decreased and humidity increased. It has been shown that low pressure stimulates the sympathetic nervous system and activates pain fibers among rats [29].

Some studies [4,30,31] have observed statistically significant effect estimates, but concluded them not to be meaningful in the clinical sense. However, even though weather effects can be small, some argue that it should not be neglected. Mukamal et al. [26] explained that implications of weather effects on ailments are different between public health and clinical perspectives. The magnitude of excess risk as a ratio as presented in our results is modest, which suggests a minimal role for the clinical setting. However, weather is ubiquitous and entire populations are constantly exposed to it, which is in contrast to the setting of potentially restricted number of individuals under specific clinical situations. Even though the effect size may appear modest in the form of a relative ratio, weather effects manifest extensively in the entire population in the form of an absolute difference. This may partially explain why the public’s belief of a weather effect on their physical health persists. Therefore, the implications of weather effects on physical symptoms in public health should not be neglected.

Stratified analyses revealed that there are differences in the perception of symptoms in response to the same weather exposure between subgroups. Women appear to be more sensitive to weather conditions than men. In general, they displayed stronger effect estimates for higher humidity and lower temperatures compared to men. This is consistent with existing studies where women have been shown to be more sensitive to pain perception than men [32]. Although the underlying mechanism is not yet fully elucidated, gonadal hormones seem to play an important role in the sensitivity of pain generating differences in pain modulation between men and women [33]. It is likely that estrogen or/and progesterone increases the threshold for pain sensitivity whereas testosterone exerts an anti-nociceptive effect [34]. Interestingly, menstrual cramps were markedly affected by lower temperature and higher humidity and both effects were statistically significant.

Many symptoms in men are associated with higher temperature, whereas women respond to both higher and lower temperatures. Women were more sensitive to higher humidity than men. Meanwhile, men appeared to be less tolerant to higher temperatures than lower temperatures.

Our study has many strengths compared to previous studies with similar topics. First, individuals were blinded to the research question by the nature of the study, and thus there was no differential information bias in reporting symptoms. In the original study [13], the participants recorded their symptoms and the type of action taken in response to the symptom. Their mindset was focused on symptoms and health care seeking behaviors, and not weather patterns. If at all, we anticipate the magnitude of such possibility is ignorable. Second, the study population was drawn from a random sample of the entire Japanese population to generate a nationally representative study population. This helped to minimize the risk of selection bias and contributed to the generalizability of study result. Compared to the majority of previous studies suffering from small sample size and lack of geographical diversity, this study benefited from including a diversity of study participants and climates, which enabled subgroup analyses. Furthermore, a longitudinal design also allowed for the control of frequent symptoms within the same individual, as well as adjusting for confounders such as socioeconomic status. 

Limitations also need to be acknowledged. Individuals were followed only for one month. Therefore, we were unable to examine the effect of weather in other seasons. Health symptoms may respond to a different range of weather conditions, such as extremely hot or cold temperatures. Results of the current analysis cannot be extrapolated to other exposure ranges and seasons. Nonetheless, October has mild temperature and climate, which offered an opportunity to examine a broad range of average weather patterns compared to other months. Still, a subsequent study with at least a one-year follow-up period covering all seasons will allow for a more thorough assessment. 

We also used monitoring stations as the source of exposure measurement. Behavioral adaptions such as use of air conditioning, humidifier/dehumidifier may modify the indoor environment. However, considering the generally mild climate of Japan and timing of the study, this feature would mitigate the magnitude of such an effect. That is, the study period was the middle of fall, where the frequency of actual use of such devices are expected to be low. Furthermore, we assessed temperature and humidity. Exposure levels to those variables would not be substantively affected by indoor environment such as precipitation. Thus, the difference between the outdoor atmosphere and the indoor air environment is expected to be low. If at all, we anticipate those discrepancies to contribute non-differential exposure measurement error, which would result in bias towards the null with wider confidence intervals [35]. Therefore, our estimation is conservative and the true effect estimate may be expected to be larger than those reported.

## 5. Conclusions

To the best of our knowledge, this is the first study of weather patterns and health to cover such a wide range of physical symptoms and weather factors that were collected in a longitudinal setting among a relatively large study population. Further study is needed to expand the time period to other seasons. The results from this study will provide scientific base that can be used towards predicting human health under certain weather conditions. Our study results can also be used to modulate the indoor environment to mitigate the symptoms of patients. For instance, one can use the air conditioner to drop the room temperature and use a dehumidifier to decrease humidity in regulating joint pain based on the present result. In doing so, age, sex, and other possible conditions should be taken into account.

## Figures and Tables

**Table 1:** Baseline characteristics of the participants in the Health Diary Study.

Characteristic	Statistic
Age, Mean (S.D.)	44.7 (23.3)
Age Group, no. (%)	
≤18 yr	801 (17.6)
>18 and <65	2680 (58.9)
≥65	1067 (23.5)
Female, no. (%)	2365 (52.0)
BMI > 25, no. (%)	722 (15.9)
Current Smoker (%)	499 (11.0)
Heavy Drinker ^†^ (%)	1055 (23.3)
Family Income, no. (%)	
≥¥10 million	475 (10.5)
≥¥4 million and <¥10 million	2836 (62.5)
<¥4 million	1225 (27.0)
Education, no. (%)	
≤Junior High	966 (21.4)
~ Associate	2305 (51.1)
≥Bachelor	1236 (27.4)

^†^ Drinks ≥ 3–4 times per week.

**Table 2:** Percent change in physical symptoms by weather.

	N	Temperature (95% CI)	Humidity (95% CI)
Joint Pains	14,099	1.87 (1.15, 2.59) *	1.38 (0.78, 2.00) *
Headache	2,899	0.56 (−0.55, 1.77)	1.35 (0.40, 2.30) *
Runny nose	4,266	0.98 (−0.03, 2.01)	−0.81 (−1.65, 0.03)
Sneeze	502	−0.21 (−2.95, 2.62)	−3.15 (−5.35, −0.91) *
Cough	3,031	−1.45 (−2.67, −0.22) *	0.14 (−0.89, 1.18)
Sore throat	1,634	−1.57 (−2.99, −0.14) *	0.12 (−1.10, 1.35)
Fever	696	2.13 (−0.13, 4.43)	0.79 (−1.11, 2.173)
Chill	154	−9.89 (−14.24, −5.33) *	−5.12 −8.59, −1.52) *
Common cold	424	−6.21 (−8.96, −3.38) *	−0.91 (−3.31, 1.54)
Muscle pain	483	4.77 (2.02, 7.59) *	0.45 −1.78, 2.73)
Backpain	527	2.35 (−0.58, 5.37)	1.02 (−1.48, 3.59)
Itchiness	995	2.48 (−0.10, 5.13)	−0.66 (−2.79, 1.51)
Eczema	358	8.54 (3.47, 13.87) *	1.33 (−2.65, 5.47)
Cramps	319	−6.00 (−8.88, −3.04) *	3.70 (1.03, 6.54) *
Fatigue	1,903	1.55 (0.09, 3.03) *	1.52 (0.28, 2.77) *
Agitation/anxiety	256	5.57 (1.05, 10.30) *	2.50 (−1.23, 6.38)
Depressed mood	175	−2.71 (−10.98, 6.33)	3.12 (−3.73, 10.45)

CI = 95% confidence interval; ^*^ Statistically significant at α = 0.05.

**Table 3:** Percent change in health symptoms associated with weather by sex.

Symptom	Men		Women	
	Temperature (CI)	Humidity (CI)	Temperature (CI)	Humidity (CI)
Joint pains	1.19 (0.06, 2.34) *	1.89 (0.92, 2.87) *	2.34 (1.41, 3.28) *	1.05 (0.26, 1.83) *
Headache	1.26 (−0.73, 3.28)	−0.16 (−1.82, 1.52)	0.28 (−1.04, 1.62)	2.04 (0.9, 3.2) *
Runny nose	0.76 (−0.69, 2.24)	−1.68 (−2.86, −0.5) *	1.18 (−0.23, 2.62)	0.04 (−1.14, 1.24)
Sneeze	0.65 (−3.68, 5.18)	−2.81 (−6.21, 0.7)	−0.75 (−4.29, 2.92)	−3.48 (−6.35, −0.52) *
Cough	−2.12 (−3.9, −0.3) *	−0.54 (−2.03, 0.98)	−0.9 (−2.55, 0.78)	0.72 (−0.69, 2.15)
Sore throat	−0.5 (−2.7, 1.74)	−1.54 (−3.37, 0.32)	−2.17 (−4, −0.3) *	1.27 (−0.34, 2.91)
Fever	3.8 (0.3, 7.43) *	−1.94 (−4.66, 0.87)	0.83 (−2.1, 3.85)	3.09 (0.44, 5.81) *
Chill	−10 (−17.18, −2.19) *	−3.66 (−9.36, 2.39)	−9.9 (−15.26, −4.2) *	−5.97 (−10.28, −1.45) *
Common cold	−4.26 (−7.93, −0.44) *	−0.62 (−3.85, 2.71)	−9.14 (−13.28, −4.81) *	−1.37 (−4.95, 2.34)
Muscle pain	5.02 (0.95, 9.26) *	−1.24 (−4.38, 2.01)	4.45 (0.76, 8.29) *	2.08 (−1.09, 5.35)
Backpain	5.49 (0.11, 11.16) *	−1.23 (−5.57, 3.31)	0.92 (−2.55, 4.51)	2.08 (−0.97, 5.21)
Itchiness	8.8 (4.67, 13.08) *	−1.18 (−4.44, 2.19)	−2.55 (−5.87, 0.89)	−0.22 (−3.01, 2.65)
Eczema	10.39 (2.38, 19.04) *	0.27 (−6.14, 7.13)	7.31 (0.87, 14.15) *	1.92 (−3.05, 7.16)
Cramps	N/A	N/A	−5.76 (−8.64, −2.79) *	3.7 (1.02, 6.45) *
Fatigue	2.91 (0.59, 5.29) *	0.49 (−1.41, 2.43)	0.52 (−1.36, 2.44)	2.29 (0.66, 3.95) *
Agitation/anxiety	−1.81 (−9.01, 5.96)	6.94 (0.18, 14.14) *	9.46 (3.67, 15.58) *	0.29 (−4.17, 4.96)
Depressed mood	10.37 (−4.97, 28.17)	4.9 (−6.9, 18.21)	−10.81 (−20.83, 0.47)	2.13 (−6.22, 11.23)

CI = 95% confidence interval; ^*^ Statistically significant at α = 0.05; N/A = not available due to N ≤ 5.

**Table 4:** Percent change in health symptoms associated with weather by age group.

Symptom	Age ≤ 18		18 < Age < 65		Age ≥ 65	
	Temperature (CI)	Humidity (CI)	Temperature (CI)	Humidity (CI)	Temperature (CI)	Humidity (CI)
Joint pains	6.87 (3.68, 10.15) *	1.52 (−1.04, 4.14)	1.36 (0.45, 2.27) *	1.56 (0.79, 2.33) *	2.56 (1.28, 3.85) *	0.96 (−0.11, 2.04)
Headache	0.11 (3.49, 3.85)	1.75 (−1.43, 5.03)	1.06 (−0.21, 2.35)	1.12 (0.05, 2.21) *	−2.81 (−5.49, −0.05) *	2.26 (−0.15, 4.73)
Runny nose	−0.19 (−1.8, 1.44)	−0.57 (−1.91, 0.79)	2.43 (0.97, 3.9) *	−1.59 (−2.76, −0.42) *	−3.56 (−6.38, −0.64) *	2.38 (−0.2, 5.02)
Sneeze	−10.83 (−16.34, −4.95) *	0.43 (−4.52, 5.62)	3.13 (−0.39, 6.77)	−5.05 (−7.71, −2.32) *	0.84 (−6.43, 8.67)	0.05 (−6.13, 6.64)
Cough	−0.84 (−2.8, 1.16)	−2.1 (−3.66, −0.51) *	−0.32 (−2.16, 1.56)	1.47 (−0.11, 3.07)	−5.91 (−8.72, −3.01) *	2.05 (−0.55, 4.72)
Sore throat	−3.31 (−6.4, −0.12) *	1.45 (−1.37, 4.35)	−0.42 (−2.16, 1.34)	−0.89 (−2.33, 0.57)	−4.17 (−7.84, −0.35) *	3.32 (−0.19, 6.94)
Fever	3.91 (−0.12, 8.1)	0.82 (−2.57, 4.32)	1.11 (−2.03, 4.36)	1.08 (−1.61, 3.85)	1.76 (−3.42, 7.21)	0.16 (−4.09, 4.61)
Chill	N/A	N/A	−8.67 (−13.83, −3.2) *	−5.9 (−10.07, −1.53) *	−15.96 (−24.14, −6.9) *	−1.99 (−8.54, 5.04)
Common cold	−10.29 (−20.45, 1.18)	−8.4 (−15.58, −0.61) *	−5.11 (−8.61, −1.47) *	−0.44 (−3.52, 2.74)	−7.96 (−12.85, −2.8) *	0.42 (−4.04, 5.09)
Muscle pain	9.03 (1.86, 16.7) *	−2.67 (−7.8, 2.74)	3.55 (0.31, 6.9) *	1.99 (−0.78, 4.84)	5.42 (−1.72, 13.08)	−2.51 (−7.86, 3.15)
Backpain	N/A	N/A	1.03 (−2.4, 4.58)	−0.32 (−3.27, 2.73)	5.44 (−0.22, 11.41)	4.26 (−0.55, 9.3)
Itchiness	8.99 (3.74, 14.51) *	−1.7 (−5.79, 2.56)	3.31 (−0.45, 7.23)	−0.4 (−3.43, 2.72)	−6.23 (−10.98, −1.23) *	−0.83 (−5.06, 3.58)
Eczema	6.73 (−0.84, 14.88)	−1.33 (−7.63, 5.4)	1.61 (−5.97, 9.79)	8.9 (1.41, 16.94) *	28.01 (13.57, 44.28) *	−5.16 (−12.27, 2.52)
Cramps	−0.79 (−10.63, 10.13)	8.17 (−2.19, 19.64)	−6.48 (−9.46, −3.39) *	3.28 (0.53, 6.1) *	N/A	N/A
Fatigue	7.95 (0.49, 15.97) *	0.41 (−5.27, 6.44)	0.83 (−0.96, 2.66)	1.8 (0.26, 3.37) *	2.31 (−0.35, 5.04)	0.99 (−1.21, 3.24)
Agitation/anxiety	N/A	N/A	5.71 (−0.1, 11.85)	0.76 (−3.74, 5.46)	5.37 (−1.94, 13.22)	5.43 (−1.31, 12.64)
Depressed mood	N/A	N/A	−0.39 (−9.63, 9.79)	2.36 (−4.89, 10.17)	−15.87 (−34.43, 7.93)	8.46 (−12.12, 33.85)

CI = 95% confidence interval; ^*^ Statistically significant at α = 0.05; N/A=not available due to N ≤ 5.

**Figure A1:**
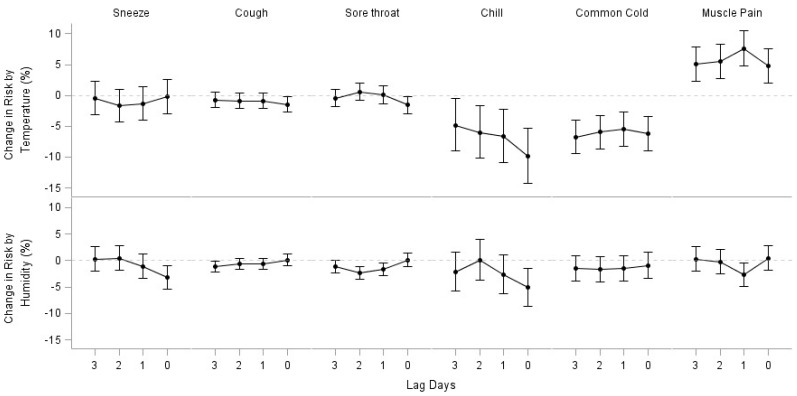
Lag Day Effects of Maximum Temperature and Humidity (sneeze, chill, common cold, muscle pain).

**Figure A2:**
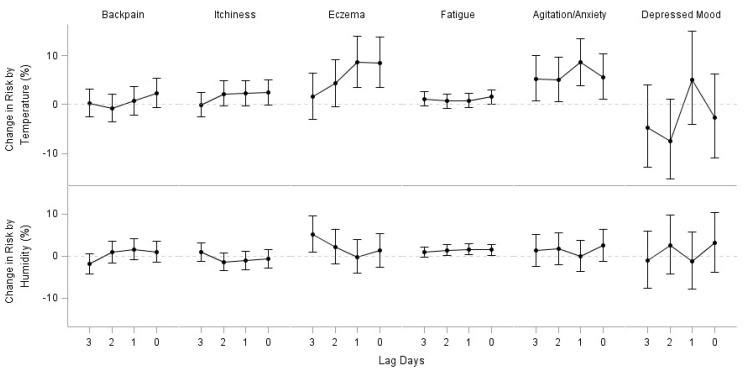
Lag Day Effects of Maximum Temperature and Humidity (back pain, itchiness, eczema, agitation/anxiety, depressed mood).
